# On the Evolutionary Trajectory of SARS-CoV-2: Host Immunity as a Driver of Adaptation in RNA Viruses

**DOI:** 10.3390/v15010070

**Published:** 2022-12-26

**Authors:** Jacob Warger, Silvana Gaudieri

**Affiliations:** 1 School of Medicine and Pharmacology, University of Western Australia, Crawley, WA 6009, Australia; 2 School of Human Sciences, University of Western Australia, Crawley, WA 6009, Australia; 3 Institute for Immunology and Infectious Diseases, Murdoch University, Mandurah, WA 6150, Australia; 4 Division of Infectious Diseases, Department of Medicine, Vanderbilt University Medical Center, Nashville, TN 37232, USA

**Keywords:** RNA viruses, SARS-CoV-2 variants, host immunity, adaptation, viral escape

## Abstract

Host immunity can exert a complex array of selective pressures on a pathogen, which can drive highly mutable RNA viruses towards viral escape. The plasticity of a virus depends on its rate of mutation, as well as the balance of fitness cost and benefit of mutations, including viral adaptations to the host’s immune response. Since its emergence, SARS-CoV-2 has diversified into genetically distinct variants, which are characterised often by clusters of mutations that bolster its capacity to escape human innate and adaptive immunity. Such viral escape is well documented in the context of other pandemic RNA viruses such as the human immunodeficiency virus (HIV) and influenza virus. This review describes the selection pressures the host’s antiviral immunity exerts on SARS-CoV-2 and other RNA viruses, resulting in divergence of viral strains into more adapted forms. As RNA viruses obscure themselves from host immunity, they uncover weak points in their own armoury that can inform more comprehensive, long-lasting, and potentially cross-protective vaccine coverage.

## 1. Introduction

The evolutionary trajectory of an organism is oriented toward the maximum likelihood for it to survive encounters with external stressors. The evolution of RNA viruses is influenced by their interaction with host immunity in much the same way. The fitness of a virus depends in part on its ability to circumvent the full artillery of host innate and adaptive immunity. A virus which reaches pandemic levels, such as SARS-CoV-2, does so by evolving into distinct viral strains, or *variants*. These variants can be characterised by specific mutations in the viral genome that may bolster evolutionary fitness—termed *viral adaptations* [1]. The frequency at which new variants emerge depends on the capacity of the virus to acquire adaptations [2,3]. RNA viruses are highly mutable and can quickly adapt to evade immune defence [2,4,5,6], which has significant bearing on clinical outcome and vaccine efficacy [7]. Mutation is sometimes associated with fitness cost [8], such that the frequency of any given mutation is often skewed in favour of mutations that impart a net fitness advantage. Understanding the pressures by which host immunity influences viral adaptation provides critical insight into the pathways utilised by RNA viruses to maximise replicative efficiency and immune evasion.

## 2. Viral Plasticity Is an Important Determinant of Fitness

Viral evolution can be thought of as a balancing act between genetic diversity and viral extinction [5,8]. The error threshold of a virus is defined as its maximum rate of mutation, beyond which mutations can result in viral failure [3,8]. This threshold is generally defined by genome length and selective constraints and represents the upper limit for even the most highly mutable viruses. 

RNA viruses tend to mutate at rates very close to this threshold [4,5,8] and replicate with relatively low fidelity compared to DNA viruses. As such, RNA viruses can diverge considerably even during a short infection [2,3,4,5,9] and generally exist in a host as a collection of genetically similar variants, termed *quasispecies* [10,11]. The ability of RNA viruses to diversify so rapidly represents a fitness advantage when infecting a biologically diverse host population [12]. For example, the spherical virion of the influenza virus is decorated by hemagglutinin glycoproteins (HA), which represent the major surface antigen. During the flu season, mutations accumulate in HA, resulting in antigenic drift [13]. As a result, the influenza vaccine is updated regularly to ensure continued protection [14]. The ability of HIV to rapidly mutate is also of significant clinical concern. The HIV genome is highly malleable and can accumulate mutation at alarming rates, such that it can rapidly develop drug resistance [9]. 

Mutations often arise during replication, and the high mutation rate of RNA viruses is generally attributable to the RNA-dependent RNA polymerase (RdRp), which is incapable of proofreading [6,15]. Coronaviruses represent one of the few RNA viruses whose viral replicase machinery has the capacity to proofread [15,16,17]. The proofreading ability of coronaviruses limits the number of genetic changes that are introduced during each round of replication, and protects its genome from excessive deleterious mutation. The coronavirus genome is composed of two large transcription units. The larger of the two units, ORF1ab, occupies more than two thirds of the genome and encodes a large polyprotein (PP1ab), which embeds 16 non-structural proteins (NSP). These proteins make up the viral replicase machinery [15,18,19]. NSP 14 represents the 3’–5’ exonuclease domain of the replicase machinery and is capable of cleaving mismatched nucleotides from the end of the polynucleotide, significantly improving replication fidelity [15,16,18,19,20,21]. As a consequence, coronaviruses have considerably larger genomes than other RNA viruses [15]. The remaining third of the coronavirus genome is mostly composed of structural proteins, including its major surface antigen—the spike glycoprotein (*S*). The low rate at which coronaviruses acquire mutations underscores those mutations that do rise to significant frequencies because they may be adaptations that afford sufficient evolutionary advantage. For example, the non-coding 5’ and 3’ terminal regions of betacoronaviruses contain various housekeeping and regulatory genes, and mutations do not accumulate in this region as they are likely to be deleterious [22,23]. Conversely, the highly immunogenic *S* protein can quickly accumulate mutations that can diminish recognition by neutralizing antibodies [24,25,26,27].

## 3. Selection Is an Indicator of Viral Evolution

Selection is an important indicator of genetic evolution and influences the frequency of particular alleles dependent on the replicative fitness of specific genotypes [28]. Indeed, the genomic landscape of SARS-CoV-2 is still actively being shaped by selection imposed by immune pressure, which may reflect the immunogenicity of various components of the virus [29]. While some parts of the genome, such as the replicase machinery, of SARS-CoV-2 are under purifying selection and are relatively conserved, positive selection is actively driving the evolution of certain regions of the viral genome [28,30]. The SARS-CoV-2 genome shows signs of continued adaptation to human immunity after transmission from the non-human reservoir, much like other zoonotic RNA viruses Middle Eastern Respiratory Syndome Coronavirus (MERS-CoV) and influenza [29,31]. Furthermore, the relative fitness of different SARS-CoV-2 variants is heterogenous across different geographic regions, likely reflecting a changing immunological landscape influenced by genetics, as well as infection and vaccination rates [30,32]. 

One way by which selection can be measured is by evaluating the relationship between the rate of non-synonymous (dN; changes amino acid) to synonymous (dS; does not change the amino acid) changes in a viral protein [1,29,33]. Shortly after the emergence of the novel Alpha (B.1.1.7) variant, we conducted a whole-genome evolutionary analysis of the SARS-CoV-2 viral genome to identify mutations occurring at ≥3% of 72,000 captured Alpha genomes from an outbreak period between 1 November 2020 and 1 February 2021 in the UK. All genomes were accessed via the GISAID public SARS-CoV-2 sequence repository, EpiCov (www.epicov.org). We used Single Likelihood Ancestry Counting (SLAC; [34]) to evaluate the dN and dS relationship at single sites across the coding regions of the genome. Our results indicate that a number of these mutations are under the influence of selection. In particular, we detected evidence of positively selected mutations in the coding regions of NSP 4 and NSP 13. A similar study conducted by Hou et al. identified both proteins as regions of strong positive selection [28]. Importantly, NSP13 embeds the viral helicase and is involved in interferon (IFN) suppression [35] pointing to immune pressure as a potential selection force. Further NSP4, in conjunction with ORF9B, is involved in extensive mitochondrial restructuring associated with severe illness, though its role in immune evasion is unclear [36].

## 4. Adaptation to Innate Interferon Signalling Can Result in Immune Suppression

**Figure 1:**
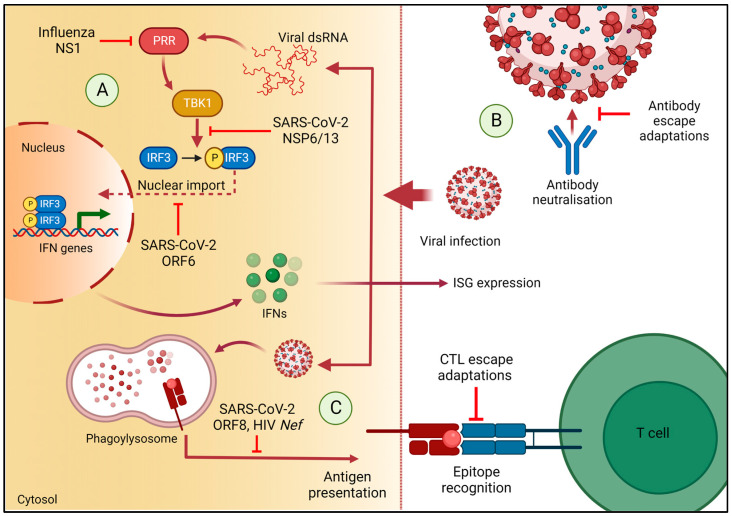
Summary of three sources of immune pressure during viral infection and viral proteins that act is immune modulators: (**A**) viral dsRNA is detected by immune sensing PRRs, triggering expression of IFN genes by phosphorylation and nuclear import of IRF-3. NS1 is a potent inhibitor of IRF-3 activation by binding viral dsRNA and preventing recognition by RIG-I. SARS-CoV-2 NSP6/13 are capable of binding TBK1 to suppress phosphorylation and activation of IRF-3, whereas ORF6 blocks its nuclear import factors; (**B**) neutralization of viral surface antigens by host antibodies, which is attenuated by adaptations in the surface antigen; (**C**) presentation of T cell epitopes by HLA class I molecules on the surface of the infected cell, which can be undermined by adaptations in HLA-restricted epitopes in a viral protein. (Created with BioRender.com).

The innate immune response is the initial call to arms, and type-I/III IFNs are the first line of defence against viral infection [37,38] (Figure 1A). Following viral entry, certain viral pathogen-associated molecular patterns (PAMPs) are recognised by host pattern recognition receptors (PRRs)—termed immune sensing. Intracellular signalling triggered by activation of PRRs leads to the transcription of type I IFNs, which go on to initiate the expression of a network of IFN-stimulated genes (ISG) to induce an antiviral state [37,39,40]. Given that this is a critical first stage of infection control, resistance to type-I IFNs provides a significant fitness advantage for RNA viruses. 

In donor-recipient HIV transmission pairs, recipient virions are uniformly more resistant to type-I IFN, indicating that the net selective pressure, which determines the transmitted founder virus, is at least partially represented by resistance to type-I IFNs [41]. This resistance is particularly important in the counteraction of IFN-β, which is a potent inhibitor of HIV-1 [42,43,44]. Furthermore, HIV-1(M), a pandemic strain of HIV, contains a number of amino acid substitutions in its capsid protein and is uniquely capable of evading immune sensing by host innate immunity. Reversing the HIV-1(M) capsid mutations results in induction of type-I IFN genes and suppression of viral replication to levels comparable to non-pandemic HIV-1, suggesting that HIV-1(M) reaches pandemic levels as a consequence of adaptations in its capsid that aid type-I IFN evasion [45].

Suppression of IFN production is a major strategy of immune evasion utilised by SARS-CoV-2. Subjects with COVID-19 exhibit vastly delayed/reduced production of type-I/III IFNs compared with individuals infected with influenza virus [46]. Furthermore, in cells infected with SARS-CoV-2, expression of IFN-β and ISG56, a family of ISGs, peaks much later compared to cells infected with Sendai virus [47]. This pattern of expression may be a result of SARS-CoV-2 viral proteins antagonising IFN production. A number of studies demonstrate that SARS-CoV-2 NSP 6 and 13, as well as ORF6, significantly inhibit activation of the IFN-β gene [47,48]. Specifically, ORF6 blocks nuclear translocation of IFN regulatory factor-3 (IRF3), a critical regulator of the IFN-β gene, by interaction with its upstream nuclear import factors [47]. Similarly, NSP 6 and 13 bind to TANK binding kinase 1 (TBK1) to inhibit phosphorylation of IRF-3 and its subsequent activation [48] (Figure 1A, Table A1).

More recent variants of SARS-CoV-2 show evidence of evolution towards IFN evasion. The Alpha variant is associated with even lower activation of IRF-3 compared to the ancestral strain, as well as reduced activation of nuclear factor kappa B (NF-κB), which is also involved in IFN-β transcription [49,50]. Upregulation of ORF6 in the Alpha variant likely also contributes to its enhanced transmission and infectivity [51,52]. Interestingly, the recently emerged Omicron subvariants BA.2 and BA.4 harbour a D61L mutation in ORF6, which attenuates its capacity to interact with IRF-3 nuclear import factors, reducing IFN evasion [52]. This mutation is no longer present in the currently circulating BA.5 subvariant. Furthermore, a nine-nucleotide deletion in NSP6 has emerged independently in several variants including Alpha, Beta (B.1.351), Gamma, and Iota (B.1.526), as well as in B lineages B.1.620, B.1.1.318, B.1.525 which may enhance IFN antagonism [53,54,55].

The SARS-CoV-2 subgenome may also play a role in IFN suppression. We previously reported that the Alpha variant produces much higher levels of SARS-CoV-2 ORF9b subgenomic RNA (sgRNA), potentially as a result of a rare triple nucleotide mutation in the nucleocapsid protein (*N*), D3L, resulting in a novel transcription regulatory sequence (TRS) upstream of the ORF9b gene [56]. ORF9b is an accessory protein, which is non-essential for virus replication, but is involved in potent type-I IFN inhibition by interaction with mitochondrial TOM70 [57]. Additionally, two adjacent R203K/G204R substitutions in the *N* protein, another IFN antagonist, result in the introduction of a TRS-like sequence motif, generating a novel sgRNA transcript [58,59]. This novel sgRNA is also present in the Gamma (P.1) and Omicron (BA.1) variants, though its precise function is unclear [51] (Table A1).

IRF-3 suppression is a common evasive manoeuvre utilised by other RNA viruses [60]. Influenza A’s non-structural protein NS1 is an important immunomodulatory protein that is capable of inhibiting IRF-3 phosphorylation and subsequent activation [61] (Table A1). The receptor binding domain (RBD) of NS1 binds to short 5’ triphosphate double stranded RNA (dsRNA) produced during infection, preventing recognition by PRRs such as RIG-I and suppressing production of IFN-β [61,62,63,64,65] (Figure 1A, Table A1). Adaptations in NS1 over time contribute to the emergence of highly virulent strains [61,66]. Isolates of the Avian influenza strain H5N1 from the 1997 pandemic harbour a P42S substitution in the RBD of NS1, which is shared with Swine influenza strain H1N1, and may enhance suppression of IRF-3 and NF-κB [66,67,68].

## 5. Repeated Exposure of Antigenic Sites to Host Adaptive Immune Memory Gives Rise to Immune Evasion

Memory B cells are responsible for the production of specific antibodies during recall of immunological memory via natural infection or vaccination. Mature memory B cells are generated in the lymphoid tissue germinal centre, where the B cell receptor undergoes immunoglobulin gene somatic hypermutation and selection in a process termed affinity maturation [69]. During affinity maturation, antibodies are selected mostly based on the strength of their affinity to the antigen, rather than their neutralizing capacity or ability to recognise closely related antigens from different viral strains [70]. This renders antibody-mediated immunity vulnerable to antigenic drift and new variants, which are sufficiently distinct from the original infectious insult, can rapidly emerge [3,70,71]. Viruses associated with acute infection are generally slow to escape adaptive immunity, because viral antigens are only transiently exposed to host immune machinery which requires prolonged exposure to influence the composition of the quasispecies [72,73]. As such, viral adaptations to the immune response tend to emerge only as the host population generates immunity [74] or during persistent infection [75]. 

Antibodies can prevent viral entry by steric hindrance of the surface glycoproteins, which are involved in cell entry (Figure 1B). Influenza’s HA is a homotrimer responsible for viral fusion via association with the sialic acid residues on host-cell membranes. The globular head of the protein (HA1) features a RBD surrounded by antigenic sites, which are targeted by neutralizing antibodies and are highly diverse between viral strains [76,77] (Table A1). This facilitates the need for regular vaccine renewal to confer sufficient protection against drifted strains. Antigenic drift also underpins the need for SARS-CoV-2 repeat vaccinations to bolster adaptive immunity against variants whose *S* genes have diverged. The *S* protein accumulates adaptations as a result of a selective drive towards infectivity and transmissibility [78,79,80], but also towards immune evasion [26]. In the early days of the SARS-CoV-2 pandemic, a prominent substitution in S, D614G, quickly reached fixation in global sequence repositories, supplanting the wild-type *S* (Table A1). This variant enhances infectivity but does not significantly alter disease severity nor attenuate neutralization by host antibodies, suggesting that it is not antigenically distinct [27,79,80]—likely because amino acid 614 lies outside the highly antigenic RBD [24,81].

Adaptations within the RBD can aid immune evasion but must do so without compromising the capacity of the virus for cell entry. As such, they are a result of competing selective pressure [24,81]. One such mutation, N439K, has arisen independently in a number of distinct variants and confers enhanced receptor binding to human ACE2 while affording protection against neutralizing monoclonal and convalescent polyclonal antibodies [25] (Table A1). This variation was one of the most prevalent *S* mutations in the global sequence repositories during 2020–2021 and is indicative of potential viral escape pathways, which are associated with little to no cost to replicative fitness. Indeed, the RBD of the Delta variant (B.1.617) exhibited considerable diversity compared to previous strains such as Alpha and Kappa (B.1.617.1), resulting in higher affinity for ACE2 and evasion of antibody neutralization; characteristics that were bolstered in the later Omicron variant [82,83,84] whose 50 mutations include 30 *S* mutations, half of which occur in the RBD [85]. Efficacy of convalescent and vaccine-induced antibodies against the Omicron variant is markedly reduced, owing to four novel mutations in the RBD [86]. Omicron sublineages BA.1.1 and BA.2, which are even more resistant to antibody neutralization, each contain novel *S* mutations [87]. 

Many *S* adaptations have arisen independently in distinct SARS-CoV-2 variants. For example, in late 2020, the *S* N501Y mutation emerged independently in a number of geographically distinct regions in several unrelated strains [53,82,88,89,90]. This mutation is shown to increase affinity for human ACE2 and is a major determinant of infection and transmission [78] (Table A1). Concerningly, the N501Y mutation emerged contemporaneously with a constellation of other mutations, including a number of additional mutations in the *S* RBD domain, which enhance receptor binding and reduce efficacy of neutralizing antibodies [91,92,93,94]. 

A number of adaptations in the *S* gene occur in its N-terminal domain (NTD) [95,96]. While the NTD was originally thought to be non-immunogenic as a result of its extensive glycan shielding [97], recent studies have identified a subset of ultrapotent monoclonal antibodies, all of which target residues within the same stretch of amino acids in the NTD—termed the NTD antigenic supersite [92,95,98]. Variants Alpha, Beta, and Delta all exhibit mutations in this site [84,92,95]. 

## 6. The Cytotoxic T Cell Response Leads to HLA-Restricted Viral Adaptation 

The cytotoxic T lymphocyte (CTL) immune response is associated with specific host human leucocyte antigen (HLA) alleles and represents a major immune pressure. HLA molecules are cell-surface proteins that present peptide fragments (epitopes) to CTLs to initiate death of the infected cell (Figure 1C). The genes that encode human HLA molecules are highly polymorphic, such that different hosts may not present the same viral targets to CTL. Thus, the HLA type of the host restricts the repertoire of viral peptides that are presented to the CTL.

HLA-restricted viral escape is a well-documented pathway for immune evasion of RNA viruses such as HIV and expression of certain HLA class I alleles is associated with lower viral load and other clinical outcomes [99,100]. During the acute phase, rapid elimination of vulnerable virus by the CTL-mediated antiviral response constitutes a selective pressure, enriching for mutations in CTL epitopes which abrogate CTL recognition and propagate over the course of chronic infection [101,102]. The Gag protein of HIV is known to generate immunodominant peptides towards which the CTL-mediated immune response is skewed [103] (Table A1). HLA-B*27 is a common allele in Caucasian populations (~8%) and is overrepresented in long-term HIV non-progressors [104]. CTL escape mutations within the HLAB*27-restricted KK10 epitope in Gag, which abrogate CTL recognition, are associated with progression to AIDS [104,105,106]. Similarly, the Hepatitis C virus (HCV) is often associated with persistent infection. During chronic HCV infection, mutations accumulate in viral T cell epitopes, which drastically attenuate T cell recognition in mutant peptides compared to the initial transmitted sequence [107]. Such mutations occur in known CTL epitopes and are associated with specific host HLA alleles [108]. For both HIV and HCV, HLA allele-associated viral adaptations have been identified across the viral genomes and account for a significant proportion of the overall diversity of these RNA viruses [109,110], and have been shown to accumulate in a host population over time [111] to become in some instances, the consensus sequence [110]. Importantly, the level of T cell-mediated viral adaptation in the autologous virus to the host’s HLA repertoire is associated with clinical outcomes [112]. 

In cases where viral fitness is undermined by CTL escape, mutations often co-occur with separate compensatory mutations. The Gag KK10 mutations impair host DNA integration and compromise the integrity of the viral capsid, significantly reducing replicative efficiency; however, this deleterious effect is rescued by a compensatory mutation in the capsid, S173A [106,113,114]. Similarly, CTL escape variant R384G in the influenza nucleoprotein obscures two CTL epitopes, HLA-B*27-restricted NP383-391 and HLA-B*08-restricted NP380-388, completely abolishing T cell recognition [115,116] (Table A1). Although this mutation quickly reached fixation in influenza strains, it is associated with a detriment to viral fitness and frequently co-occurs with a number of compensatory mutations [117,118]. 

The extent to which CTL-mediated immunity exerts an evolutionary force on SARS-CoV-2 remains contentious. The SARS-CoV-2 ORF8 protein, which shares the least homology with other coronaviruses, is capable of down-regulating HLA class I molecules, impairing CTL-mediated immunity [119], a characteristic shared by HIV’s Nef protein [120] (Figure 1C, Table A1). Nevertheless, a number of CTL epitopes derived from SARS-CoV-2 viral proteins have been identified [121]. Motozono et al. describe a 9-mer peptide in the RBD of the SARS-CoV-2 *S* protein, NF9, which is shown to be an immunodominant epitope presented by common HLA-A alleles [122]. The same study established that SARS-CoV-2 variants such as variant Epsilon (B.1.427/429) and B lineage B.1.1.298 can evade HLA-restricted CTL detection and show significantly enhanced infectivity and virulence. Furthermore, SARS-CoV-2 variants Alpha, Beta, Gamma and Delta all harbour mutations in four known HLA-A-restricted CTL epitopes in *S* and ORF1a, which significantly attenuate CTL activation [123]. However, it is difficult to determine whether these mutations are the result of CTL adaptation or other immune pressures acting on the *S* protein [124]. Many SARS-CoV-2 CTL epitopes occur in internal proteins, which are more conserved and are can consistently elicit a CTL response across several distinct variants [125,126]. CTL escape may not be as extensive for SARS-CoV-2 as observed for the more mutable RNA viruses such as HIV and HCV that are also associated with chronic infection [127]. 

## 7. Chronic Infection May Accelerate Viral Adaptation

The global SARS-CoV-2 pandemic can be characterised by the periodic emergence of highly divergent viral variants. Such punctuated evolutionary shifts, termed saltational evolution [128], are not characteristic of stepwise diversification expected from multiple host-host transmissions. Most SARS-CoV-2 infections are acute, and de novo intrahost viral diversity is relatively low [129]. The infection event constitutes a bottleneck, resulting in the transmission of a small number of founding viruses from the donor viral pool. This founding virus is usually a representative of the dominant viral strain from the infectious donor, which frequently corresponds to the dominant circulating strain at the time [75]. 

Mounting immune pressure over the course of persistent infection can induce viral adaptation—thus, intrahost viral diversity may be considered a function of infection time. Accordingly, the likelihood of a transmitted founder virus harbouring de novo variation increases as infection persists [75]. In cases of incomplete immunity experienced by subjects with primary or acquired immunodeficiencies, or undergoing immunosuppressive therapy, viral clearance is prevented as compared to immunocompetent subjects [130]. As a consequence, replicating virus can persist in the host for longer periods of time. The chronic infection hypothesis of SARS-CoV-2 viral divergence posits that highly divergent viral variants evolve in such subjects before transmitting into the general population [131]. Such mutational jumps are relatively understudied in the context of human viral disease. Nevertheless, several studies report cases of punctuated, extensive adaptation in viral isolates from cases of persistent infection [128,131,132,133,134,135,136,137].

A case study published by Avanzato et al. documents persistent infection of a septuagenarian female experiencing immune dysfunction as a result of acquired hypogammaglobulinemia [136]. The subject was admitted in early 2020 with SARS-CoV-2 infection which persisted for 105 days and was treated with two doses of convalescent plasma therapy. Genomic sequencing of viral isolates from day 70 of infection uncovered a 12-nucleotide deletion resulting in deletion of amino acids 141–144 in *S* in 100% of reads. This deletion falls within the NTD antigenic supersite [96,98,136] and overlaps a 3-nucleotide deletion (Δ143-145) observed in Omicron [137] (Table A1). A similar case study describes a septuagenarian male who was experiencing severe combined immunodeficiency and was admitted for persistent SARS-CoV-2 infection [134]. Treatment with convalescent plasma therapy yielded dramatic punctuated shifts in viral diversity towards escape mutants, including deletions in the NTD (Δ69/70), as well as a D796H substitution in the S2 subunit [134] (Table A1). These variations were less sensitive to antibody neutralization. Of note, Δ69/70 has been documented in several distinct and independent lineages and may be an example of evolutionary convergence [53,138,139]. 

A third study published by Cele et al. describes a subject experiencing persistent SARS-CoV-2 infection as a result of immune suppression associated with HIV infection. This study documents the de novo emergence of adaptations in the RBD, including adaptations which reduce efficacy of Delta neutralizing antibodies despite infection time predating the emergence of the Delta variant [137]. 

The overall mutational patterns observed in chronic SARS-CoV-2 infection often correspond to those observed in emerging variants of concern, however in many cases, such intrahost viral evolution is host-specific and divergent virions often do not carry key mutations observed in circulating variants. Harari et al. report that mutations associated with higher transmissibility, that are documented in circulating variants, are not detected in high numbers in chronically infected hosts [140]. The authors suggest that there may be insufficient pressure from the chronically infected host to drive evolution of more transmissible variants, rather intrahost viral adaptation is skewed in favour of replicative fitness that is bolstered by immune evasion [140]. 

## 8. Cross Protective Immunity Highlights Conserved Targets for Immunisation

While broad immunity is often thwarted by antigenic drift, repeated exposure to the same or similar viruses can provide valuable insight into those peptides that represent a more suitable target; peptides that are antigenic and functionally indispensable. Antibodies against these conserved peptides often impart a broad range of immunity against drifted strains. Such viral cross reactivity can inform the development of putative universal vaccines. The HA protein of the influenza virus is its most highly variable protein and represents a moving target for influenza immunisation, as such, prospective universal influenza vaccines should also target more conserved regions [141]. The membrane proximal stalk domain of HA, which is involved in several structural rearrangements to facilitate internalisation of the influenza virus into the host cell, is functionally indispensable and mutations in this protein are usually not tolerated [142,143]. This region is therefore relatively resistant to antigenic drift, representing an attractive target for universal influenza vaccine design [144]. In support of this approach, anti-stalk antibodies can provide protection against several distinct influenza strains and accumulate after repeated infection [13,14,143].

The emerging SARS-CoV-2 Omicron variant and its sublineages harbour adaptations in previously conserved RBD residues, which aid in evasion even of cross-protective antibodies with broad neutralization capacity against other sarbecoviruses [145,146,147]. As such, determining a suitable target for vaccination that can protect against emerging variants is of significant interest. Pre-existing immunity for SARS-CoV-2 has been described in human hosts naïve to SARS-CoV-2 [148]. Convalescent SARS-CoV subjects retain robust cross-reactive memory CD4+ T cells against the SARS-CoV-2 *N* protein up to 11 years after infection [149,150]. *N* is the most abundant protein in the SARS-CoV-2 proteome and maintains a high degree of homology between a number of beta coronaviruses [149]. Furthermore, *N* contains an immunodominant T cell epitope recognised by SARS-CoV- and MERS-CoV-infected HLA-DR2 and -DR3 transgenic mice, and provides protection against bat CoVs, which may be an attractive target for pan-coronavirus heterologous immunisation. Interestingly, when comparing CD4+ T cell reactivity in SARS-CoV-2 naïve individuals with historic exposure to the common cold coronaviruses, cross-reactivity against a number of SARS-CoV-2 specific peptides was observed, including NSP14, NSP4 and NSP6, but only marginally against *N* [148].

## 9. Conclusions

RNA viruses’ considerable capacity for adaptation represents the ever-present threat of pandemic viral outbreak of which SARS-CoV-2 is an emphatic reminder. The emergence of a pandemic RNA virus is followed by an evolutionary arms race wherein host immunity must mount a successful defence faster than the virus can adapt. It is critical to understand the vulnerabilities of the RNA virus, which are inevitably influenced by host immunity, in order to mitigate its impact on the global community and inform comprehensive vaccine coverage and pre-empt its evolutionary trajectory. 

Adaptation to the innate IFN response represents a critical turning point for an infecting virus. A number of RNA viruses have evolved the capacity to down regulate IFN regulatory elements. Emerging SARS-CoV-2 variants have a similar effect on expression of IFN-β through interaction with IRF-3 as do seasonal influenza viruses, effectively sequestering it and preventing transcription of its downstream genes. This capacity for IFN evasion is renewed with the emergence of adaptations in new variants and highlights the importance of IFN-β regulation in control of infection.

Adaptation to humoral immune memory represents a delicate balancing act wherein the viral surface proteins are frequently the subject of immense selective pressure. Influenza’s globular HA domain is its most antigenic asset and can acquire mutation at little-to-no cost to replicative fitness. As such, it can quickly acquire antibody escape characteristics. Similarly, mutations in a SARS-CoV-2 antigenic supersite are the result of considerable immune pressure in a region which is highly exposed and dispensable to function, resulting in escape mutations in this region, which rise in frequency as new variants emerge. Such adaptations can also emerge de novo during persistent infection. This phenomenon is popularly cited as the evolutionary mechanism by which highly divergent SARS-CoV-2 variants first emerged in the host population.

CTL immunity represents a host-specific selective force on many RNA viruses. Such HLA-restricted viral escape is well documented in the context of HIV and HCV but remains controversial in the case of SARS-CoV-2. While a number of variants harbour mutations in known HLA-A restricted CTL epitopes, other epitopes lie within relatively conserved proteins and CTL escape may be limited. However, the importance of CTL immune responses against SARS-CoV-2 is highlighted by the downregulation of HLA class I by the ORF8 protein. 

As the viral genomic landscape changes, it becomes clear precisely which genomic regions cannot tolerate extensive mutation. Such viral proteins as influenza’s membrane proximal Hemagglutinin stalk or the *N* protein of SARS-CoV and SARS-CoV-2 are suitable candidates for universal immunisation because they are antigenic but functionally indispensable and demonstrate a resilience to evolutionary change that can be relied upon with some degree of certainty.

As the global community begins to emerge from the SARS-CoV-2 pandemic, continued monitoring of emerging variants is critical to better understand the weak-points of the RNA virus and pre-empt future viral outbreaks.

## Appendix A

**Table A1:** Summary of viral proteins involved in defence against host immunity for various RNA viruses, some of which show evidence of adaptation towards viral escape (examples provided).

SARS-CoV-2			
**Coding Region**	Role in Viral Defence	Adaptations	Consequence
**NSP6/13**	IFN antagonism (TBK1 inhibition) [48]	9 nucleotide deletion [53]	
**ORF6**	IFN antagonism (blocking of IRF-3 nuclear import factors) [47]	D61L [52]	IFN susceptibility
**Nucleocapsid**	IFN antagonism	R203K/G204R [58]	Novel subgenomic transcript
		D3L [56]	Upregulation of ORF9b
**Spike**	Surface antigen	D614G [27]	Increased infectivity
		N439K [25]	Increased infectivity, antibody evasion
		N501Y [53]	Increased infectivity, antibody evasion
		NTD Δ143-145 [136]	Antibody evasion
		NTD Δ141-144 [137]	Antibody evasion
		NTD Δ69/70 [134]	Antibody evasion
		S2 D796H [134]	Antibody evasion
**ORF8**	HLA-I suppression		CTL escape
**Influenza**			
**NS1**	IFN antagonism (sequestration of viral dsRNA) [61]	P42S [67]	IFN evasion
**Nucleoprotein**	Encapsulates viral genome	R384G [115]	HLA-*27/08 restricted CTL escape
**HA**	Surface antigen		Antibody evasion
**HIV**			
**Gag**	Immunodominant CTL target	HLAB*27 restricted KK10 epitope mutations [106]	CTL escape
**Capsid**	Encapsulates viral genome	S173A [106]	Compensatory to Gag KK10 escape mutations
**Nef**	HLA-I suppression		CTL escape
